# “*Whatever journey you want to take, I’ll support you through*”: a mixed methods evaluation of a peer worker program in the hospital emergency department

**DOI:** 10.1186/s12913-023-10532-5

**Published:** 2024-01-30

**Authors:** Meghan O’Neill, Camilla Michalski, Kate Hayman, Jennifer Hulme, The Neighbourhood Group Community Services(Lorie Steer), Florencia Leston, Amber Kellen, Lorie Steer, Sané Dube, Lori M. Diemert, Kathy Kornas, Alice Schoffel, Laura C. Rosella, Andrew Boozary

**Affiliations:** 1 Population Health Analytics Lab, Dalla Lana School of Public Health, University of Toronto, Toronto, ON Canada; 2 University Health Network, Toronto, ON Canada; 3 Department of Medicine, University of Toronto, Toronto, ON Canada; 4 Department of Family and Community Medicine, University of Toronto, Toronto, ON Canada; 5 Gattuso Centre for Social Medicine and Population Health, University Health Network, Toronto, ON Canada; 6 Laboratory Medicine and Pathobiology, Temerty Faculty of Medicine, University of Toronto, Toronto, ON Canada; 7 ICES, Toronto, ON Canada; 8 Institute for Better Health, Trillium Health Partners, Mississauga, ON Canada; 9 Institute for Health Policy Management and Evaluation, University of Toronto, Toronto, ON Canada

**Keywords:** Peer worker, Harm reduction, Hospital-based care, Mixed-methods

## Abstract

**Background:**

People who are unhoused, use substances (drugs and/or alcohol), and who have mental health conditions experience barriers to care access and are frequently confronted with discrimination and stigma in health care settings. The role of Peer Workers in addressing these gaps in a hospital-based context is not well characterized. The aim of this evaluation was to 1) outline the role of Peer Workers in the care of a marginalized populations in the emergency department; 2) characterize the impact of Peer Workers on patient care, and 3) to describe how being employed as a Peer Worker impacts the Peer.

**Methods:**

Through a concurrent mixed methods evaluation, we explore the role of Peer Workers in the care of marginalized populations in the emergency department at two urban hospitals in Toronto, Ontario Canada. We describe the demographic characteristics of patients (*n* = 555) and the type of supports provided to patients collected through a survey between February and June 2022. Semi-structured, in-depth interviews were completed with Peer Workers (*n* = 7). Interviews were thematically analyzed using a deductive approach, complemented by an inductive approach to allow new themes to emerge from the data.

**Results:**

Support provided to patients primarily consisted of friendly conversations (91.4%), discharge planning (59.6%), tactics to help the patient navigate their emotions/mental wellbeing (57.8%) and sharing their lived experience (50.1%). In over one third (38.9%) of all patient interactions, Peer Workers shared new information about the patient with the health care team (e.g., obtaining patient identification). Five major themes emerged from our interviews with Peer Workers which include: (1) Establishing empathy and building trust between the patient and their care team through self-disclosure; (2) Facilitating a person-centered approach to patient care through trauma-informed listening and accessible language; (3) Support for patient preferences on harm reduction; (4) Peer worker role facilitating self-acceptance and self-defined recovery; and (5) Importance of supports and resources to help Peer Workers navigate the emotional intensity of the emergency department.

**Conclusions:**

The findings add to the literature on Peer Worker programs and how such interventions are designed to best meet the needs of marginalized populations.

**Supplementary Information:**

The online version contains supplementary material available at 10.1186/s12913-023-10532-5.

## Background

Despite Canada’s universal health care system, people who are unhoused, have substance use (drugs and/or alcohol) and have mental health conditions (defined hereafter as marginalized populations), often face social and structural barriers to receiving health care [1–3]. This population also experiences higher levels of substance use morbidity and exhibits higher emergency department (ED) use than the general population [4–7]. In North America, ED visits for substance use related conditions have increased substantially, [8, 9] as has mortality from drug overdoses [8, 10–14]. In Ontario, Canada, a recent population-based study found that ED visits for drug overdoses grew during the re-opening periods of the COVID-19 pandemic among recently homeless populations compared to those with stable housing [15]. Additionally, income-based disparities in ED visits due to alcohol use have increased over the same time [16].

Despite higher ED use and need for care, abstinence-based drug policies and poor pain management protocols contribute to marginalized populations leaving the hospital on their own decision, cyclically resulting in treatment delays and increased mortality risk [17–19]. Length of stay is often higher for unhoused populations as a result of long wait times to secure a shelter bed and discharge policies that are not designed for patients without a fixed address [20]. This dynamic is playing out on the backdrop of discrimination and stigma [21–23] that manifest at the interpersonal (e.g., provider and patient interactions), organizational (e.g., institutional policies), and structural levels (e.g., laws). These levels of discrimination and stigma perpetuate fear of hospital-based settings and results in poor care experiences for marginalized groups.

Peer Workers, otherwise known in the literature as Peer Support Workers, Peer Helpers, or Natural Helpers, are individuals, “…with equal standing within a particular community who share a common lived experience” [24]. Integrating Peer Workers into health care teams in a variety of settings reduces barriers to accessing care and improves care experiences [25]. The proposed mechanism is through the ability of a Peer Worker to foster equally-shared power and a harm-reduction approach with a patient where the relationship serves as the foundation for empowerment and engagement [26]. Peer Workers in health care settings successfully address the lack of trust towards health care providers (HCPs), increase the likelihood of future care-seeking, and counteract negative perceptions of health care [27, 28]. Community-based Peer Workers have also succeeded in linking patients to care [29, 30] and in supporting harm reduction education [31, 32].

Despite gaps that persist for marginalized populations in hospitals, few studies have reported on the impact of Peer Workers in this setting. To date, three studies have reported on Peer Worker interventions for people who use drugs in an in-patient substance use treatment setting [33–35]. These studies focused on people who use drugs and did not explicitly consider the intersection of structural vulnerabilities such as being unhoused in the ED. The aim of this evaluation was to 1) outline the role of Peer Workers in the care of a marginalized populations in the emergency department; 2) characterize the impact of Peer Workers on patient care; and 3) to describe how the role of a Peer Worker impacts the individual, both personally and professionally.

## Methods

### Study setting and design

This study reports on a concurrent mixed methods evaluation of the Peer Support in the ED Program, which takes place at two high-volume urban hospitals in Toronto, Ontario Canada. Combined, these two hospital EDs serve approximately 120,000 patients per year [36]. The evaluation was designed with formative and outcome orientated objectives. Specifically, formative evaluation questions included:

1. How many, and what were the characteristics of, patients seen by Peer Workers?
2. What social supports and services were provided to patients supported by a Peer Worker?

Outcome oriented questions primarily focused on the extent to which the program is achieving early outcomes, including:

1. How, if at all, do Peer Workers impact the provision of care to patients during their ED visit?
2. How has the Peer Worker role impacted the Peer, both personally and professionally?

The Peer Support in the ED Program was launched in response to the COVID-19 pandemic in 2020, the subsequent rise in marginalized populations seeking care in the ED, and the urgent need to improve care delivery and available supports for these patients. The program was born out of existing collaborations, reciprocal trust and a shared vision between the University Health Network (the hospital) and The Neighbourhood Group (community-based partner). A trusted community partner that is skilled at training and supporting Peer Workers was integral to the program’s success and sustainability. Peer support programs are often integrated into a hospital-based setting in one of two ways: the hospital partners with the community-based organization in program delivery, or the hospital employs Peer Workers directly [37]. The program described here utilizes the partnership model in which the hospital and the community-based partner established a memorandum of understanding.

Peer Workers were scheduled in pairs for an eight-hour shift in the ED, and worked in collaboration with other HCPs such as social workers, nurses, and doctors with direct oversight provided by a manager from The Neighbourhood Group. The Peer Workers in the ED Program operated on a turnover system whereby all Peer Workers are employed for a short term (typically a 6-month contract). Their responsibilities included, but were not limited to: building rapport with patients; listening and communicating their lived experience to support the patient (i.e., experiences related to being unhoused, having a mental health condition, and/or substance use (drugs and/or alcohol)); supporting the patient in meeting their basic needs (e.g., providing clothing and food); providing assistance with navigating social service resources (e.g., harm reduction supports); and conflict de-escalation, where appropriate.

This evaluation used principles of a community-based participatory approach to research, in that the evaluation design, implementation, and interpretation of results were informed by the first-hand expertise of the program’s planners and front-line providers [38, 39]. The primary avenue for participation involved an Evaluation Advisory Group composed of (1) representatives from the University Health Network ED including nursing managers and physicians; (2) senior leadership and managers of the Peer Workers at The Neighborhood Group, including former Peer Workers; (3) managers and program coordinators for the Gattuso Centre for Social Medicine at UHN; and (4) members of an external evaluation team from the University of Toronto. These groups met bi-weekly to determine the priorities of the evaluation, create data collection instruments, facilitate the recruitment of participants, and provide feedback on preliminary results. The evaluation team also liaised with one former and one current Peer Worker at key points throughout the evaluation to solicit feedback on data collection tools and interpretation of results.

Quantitative and qualitative information were collected during the same time frame, analyzed independently, and then combined to enrich our understanding of the Peer Workers in the ED Program [40]. This model was chosen because it afforded us deeper and more nuanced answers to our evaluation questions, was appropriate for the population under inquiry (i.e., marginalized populations), and allowed us to answer evaluation questions that could not be adequately answered by quantitative or qualitative data alone. The primary sources of data were a Patient Interaction Survey completed by Peer Workers and semi-structured interviews conducted with Peer Workers.

### Patient interaction survey

The Patient Interaction Survey is a secure data collection instrument stored in the Research Electronic Data Capture (REDCap) system that the Peer Worker completed once a patient they supported was discharged or once they completed their shift. The survey was co-designed by Evaluation Advisory Group and was in use at both program sites from February 4th, 2021 to present. As part of the documentation process, Peer Workers received an orientation to data entry in the Patient Interaction Survey. The survey is not connected to the electronic patient record and includes de-identified patient information entered by the Peer Worker. Information collected in the Patient Interaction Survey includes demographic information (i.e., age group and gender), the types of substances the patient disclosed that they have used, the types of resources and supports offered to the patient (e.g., arranged for a shelter bed, connected patient with harm reduction supports), and patient outcomes (e.g., whether the patient followed their plan of care or whether the patient left on their own decision)(See Additional file 1).

### Interviews

Semi-structured, in-depth interviews were conducted in October 2021 with seven Peer Workers who were purposively selected [41] because they were employed by the Neighbourhood Group and currently or previously worked at one of the two program sites. The interview guide included questions about their experiences interacting and communicating with patients; communicating with other HCPs; navigating different perspectives among the health care team; their perceptions on how they impact the provision of care; and how being a Peer Worker may have impacted them, both personally and professionally (See Additional file 2).

Participation in the interviews was confidential and voluntary. Informed verbal consent was obtained from participants. Interviews were audio recorded and transcribed verbatim using a professional transcription service. On average, interviews lasted between 40 and 60 min and were conducted via phone or video call. Peer Workers participated during work hours and were compensated at their hourly rate.

### Data analysis

Quantitative data on patient characteristics from the Patient Interaction Survey were analyzed descriptively using SAS (Version 9.4) for all patients seen by a Peer Worker. Interview transcripts were thematically analyzed using both deductive and inductive strategies to ensure a thorough exploration of the data in alignment with our evaluation framework [42]. In the deductive phase of our analysis, we followed a structured approach by initially developing a preliminary codebook a priori. This codebook was carefully designed to reflect the key evaluation questions that had guided the construction of our interview guide. It served as our foundation for systematically categorizing and synthesizing the qualitative data. Simultaneously, an inductive approach was applied to allow themes to organically emerge from the data. This more open-ended exploration allowed us to capture unanticipated insights and nuances within the data that might not have been accounted for in our predefined codebook.

Qualitative analysis proceeded as follows: two authors (M.O., C.M.) coded the same transcript together. The authors then reviewed the codes, discussed new and emergent themes, and subsequently refined the codebook. The two authors independently applied the codebook to a second transcript and exchanged their analysis to review points of convergence and divergence. Finally, the remainder of the transcripts were divided and coded independently, with meetings held on an ad-hoc basis to refine themes and discuss saturation. Throughout the interviews, reflexivity and positionality was discussed and documented in the form of note taking and memos.

Thematic analysis was conducted using *NVivo* (QSR International, Version 12). To increase the credibility and trustworthiness of our analysis, we returned our summary to two Peer Workers as a form of respondent validation. The Peer Workers were asked to provide feedback on whether our summary was consistent with their experiences and was subsequently updated and finalized according to their feedback. The qualitative analysis presented in this evaluation is reported in line with the Consolidated Criteria for Reporting Qualitative Research checklist [43].

## Results

In total, Peer Workers provided support to 555 patients in the ED from February 4th- June 30th, 2022. Most patients supported by Peer Workers were male (66.7%) and more than half of patients (54.6%) were between the ages of 30–49 years old (Table 1). Alcohol was the most reported substance used by patients (25.5%), followed by opioids (12.8%) and stimulants (15.0%). Peer Workers supported an average of six patients per eight-hour shift.

**Table 1:** Characteristics of patients seen by Peer Workers in the emergency department from February 4th- June 30th, 2022 (*N* = 555)

Characteristic	N (%)
**Gender**	
Man (cis, trans)	370 (66.7%)
Woman (cis, trans)	185 (33.3%)
**Age group**	
Less than 29	105 (18.9%)
30–39	179 (32.3%)
40–49	124 (22.3%)
50–59	73 (13.2%)
60 and up	74 (13.3%)
**Substance use**^**a**^	
Alcohol	141 (25.5%)
Opioids (e.g., opioids, sedatives, benzodiazepines, etc.)	71 (12.8%)
Stimulants (e.g., methamphetamine, cocaine, etc.)	83 (15.0%)
**Number of patients by month**	
February 2022	127 (22.9%)
March 2022	117 (21.1%)
April 2022	110 (19.8%)
May 2022	113 (20.4%)
June 2022	88 (15.9%)
**Average number of patients supported per shift (mean (range))**	5.50 (1–14)

^a^Options are not mutually exclusive. Multiple response options may apply to a single patient

The three most common referrals provided to patients by Peer Workers included referring patients experiencing homelessness to a shelter bed (54.1%), connecting patients to a Rapid Access Addiction Medicine (RAAM) clinic, management withdrawal services clinic, detox/rehab centre, or other substance use support services including treatment (35.0%), and connecting patients to a hot meal or food security/meal site (23.4%) (Table 2). The most common tangible items provided were food (86.1%), transportation funds (48.3%), clothes (35.9%), Naloxone kits (3.6%), and other harm reduction supplies (1.8%).

**Table 2:** Summary of resources and supports provided to patients seen by Peer Workers in the emergency department from February 4th- June 30th, 2022 (*N* = 555)

Characteristic	N (%)
**Service referrals provided to patients**	
Found patient a shelter bed	300 (54.1%)
Connected patient to a rapid access addiction medicine clinic, detox/rehab centre, or other addiction support group	194 (35.0%)
Referred patient to a hot meal or food site	130 (23.4%)
Connected patient to a mental health treatment centre	28 (5.0%)
Connected patient to other treatment centre (e.g., community based primary care clinic)	11 (2.0%)
Longer-term housing support (e.g., help with housing application)	8 (1.4%)
Call to crisis hotline (e.g., domestic violence)	7 (1.3%)
Supported patient with income aid (e.g., Ontario Works application)	4 (0.7%)
Supported patient in finding employment	3 (0.5%)
Connected them with their TNG case worker	1 (0.2%)
Other supports	18 (3.2%)
**Tangible resources offered to patients**	
Food	478 (86.1%)
TTC tokens/taxi transport	268 (48.3%)
Clothes	199 (35.9%)
Naloxone kit	20 (3.6%)
Other harm reduction supplies (e.g., long needles)	10 (1.8%)
Other resources	15 (2.7%)
Emotional support provided to patients^*^	
Had friendly & empathetic conversations	507 (91.4%)
Supported discharge planning	331 (59.6%)
Helped navigate their emotions/mental wellbeing	321 (57.8%)
Shared lived experience	278 (50.1%)
Advocated to hospital staff for patient care	87 (15.7%)
Brought them out for a cigarette break	59 (10.6%)
Provided information on hospital resources or what will be happening to them in the ED	37 (6.7%)
Other	13 (2.3%)
**Instances where the peer shared new information about the patient with other health care providers**	216 (38.9%)
**Number of successful conflict de-escalations**^**b**^	40 (60.6%)

^*^Please note that options are not mutually exclusive. Multiple response options may apply to a single patient

^b^A working definition of de-escalation was provided to Peer Workers as a reference. De-escalation was defined as efforts to decrease the intensity or seriousness of a situation (e.g., calming down a visibly angry or violent patient)

Interactions between Peer Workers and patients primarily consisted of friendly conversations (91.4%), supporting discharge planning (59.6%), providing tactics to help the patient navigate their emotions/mental wellbeing (57.8%), and sharing their lived experience (50.1%). In over one third (38.9%) of all patient interactions, Peer Workers were able to share new information (i.e., not already recorded in the patients’ medical record) about the patient with the health care team with the explicit consent of the patient. Examples include confirming the patient’s identification, uncovering what happened to the patient before they presented in the ED, or identifying what types of substances they may have used prior to their ED visit. Among the 555 patient interactions logged by the Peer Workers, 66 (11.9%) met the criteria for an escalating situation with a patient, defined as a circumstance in which the intensity, seriousness, or potential for conflict, harm or adverse outcomes is growing. This type of scenario typically involves a progressive or rapid worsening of a situation, characterized by heightened emotions, tension, or aggression among individuals involved. Among those patients, Peer Workers were able to help de-escalate more than half (60.6%) of situations.

### Role overview: investigative and advocative function

In Peer Worker interviews, the role was often presented as filling a gap in the biomedical care model, usually in reference to compassionately engaging with a patient’s humanity and acknowledging their broader life context. The role was encapsulated as having two primary functions: investigative and advocative. Regarding the first, the Peer Workers championed their lived experience as setting precedent for open and communicative interactions with patients. The information gained during these interactions was then used to support and advocate for the patient. Generally, Peer Workers described a typical interaction with a patient as disclosing their own lived experience and a description of their functional role (i.e., the types of supports and resources they could provide). Sharing these two pieces of information helped the peer team to set a precedent of non-judgmental care in terms of information exchange with patients, and of transparency and reliability in terms of the scope of their role.

#### Theme 1: establishing empathy and building trust between the patient and their care team through self-disclosure

Several Peer Workers referenced a rooted distrust between patients and HCPs, citing their own and patients’ past negative experiences. They referenced their own and patients’ feelings of stigma-induced shame, disjointed and sterile care, atmospheres of condescension, and having disparate life circumstances as a few of the driving forces behind this distrust: *“Most of the time they don’t really feel comfortable talking to doctors and nurses just because they have a preconceived notion that the doctors and nurses look down on them just because they are an addict” [Peer 2].*

Many Peer Workers highlighted the ways in which their lived experience and non-clinical backgrounds set them apart from other HCPs, which can be a remarkable distinction for patients in terms of trust-building: *“I kept saying to the patient, ‘I fully understand what you’re going through’… Once he heard that word, ‘I fully understand what you’re going through’ it’s like, he tuned in… truthfully all they want to know is that somebody is not going to judge them… they just want to know that the person that they’re going to disclose their information to is not going to turn around and use that information against them or ill treat them or look down on them because of this illness” [Peer 6].*

Disclosing their lived experience was so effective in helping build rapport that it was often disclosed early on, as they introduced themselves to patients. One peer viewed sharing their lived experience as an automatic translation of empathy, which built rapport and trust in ways that sympathy alone could not: *“It makes them a lot more comfortable and then they open up and they tell you what’s going on… you know it’s not one of those, ‘Oh, that must be so terrible’” [Peer 2].* A critical component of translating this sense of empathy is having the same lived experience as the patient, and as such, Peer Workers highlighted the importance of having a diverse variety of lived experience across the team. When sharing their past, Peer Workers noted they are careful not to make the patient feel as though they are making assumptions about their histories. As such, Peer Workers restricted this disclosure to themselves, and were careful not to allude to or probe the patient for similar histories. Often, their own disclosure was enough to elicit a more receptive and trusting demeanour on behalf of the patient.

While the Peer Workers aimed to build trust in the space between patients and themselves, they also facilitated trust-building in the space between patients and other HCPs. One Peer Worker facilitated this by expressing their own positive opinion of other HCPs, in this case, a nurse: *“There’s certain patients who make it very clear that they don’t like anybody so [I] always try to pick at least one nurse every time they come and say, ‘No, this person is cool, don’t worry, they’re good’” [Peer 2].* Following conversations regarding patients’ substance use, this same Peer Worker provided an example in which they encouraged patients to further share this information with the health care team, *“[The patient] was telling me everything and I said, ‘Hey, why don’t you let [the nurse] know what’s going on with you. That’s the only way she can better assist you,’ so I brought the nurse in…” [Peer 4].*

#### Theme 2: facilitating a person-centered approach to patient care through trauma-informed listening and accessible language

The importance of trauma-informed listening was highlighted as central to communicating with patients and necessary for providing person-centered care. Peer Workers made sure to (1) relieve pressure to share information and (2) maintain neutrality when listening to and advocating for a patient’s desired care resourcing. Regarding the first point, in a similar way that Peer Workers emphasized the importance of not assuming a patient’s lived experience when disclosing their own, Peer Workers empowered the patients to dictate how much or how little they wanted to share: *“[I] try to be very careful not to make them re-tell their story because it brings up more trauma… You don’t try to probe and figure out what happened to them. You let them speak…when they’re ready to speak and if they’re ready to speak” [Peer 1]*.

Peer Workers brought awareness to the re-traumatizing experience of having to repeat personal histories to different HCPs: *“I always imagine how I would feel… what would I want them to avoid asking me… I feel like as soon as [the patients] come in, [they’re] asked on the phone when they’re calling 9–1-1, ‘What happened?’ then when they get in the ambulance, ‘What happened?’ then when they get to triage, ‘What happened?’ then when they get to the nurses, ‘What happened?’ then when they get to the doctor, like it’s [a] continuous ‘What happened, what happened, what happened?’ and they have to retell the story so many times and that’s pretty triggering” [Peer 2].* To relieve the pressure to revisit the potentially traumatizing histories, one Peer Worker shared their tactic of starting off interactions with offers of food, clothing, or blankets – no questions asked.

Just as Peer Workers honoured patient’s reluctance to share stories, Peer Workers also honoured the therapeutic process of feeling heard. A few Peer Workers recalled having lengthy conversations with patients on topics spanning far beyond the scope of the patient’s ED stay. At least two Peer Workers mentioned that even when patients were seemingly talking about unrelated issues, actively listening was key because important information can be revealed.

Another peer contrasted the response of Peer Workers knowledge of current substance use to what may be heard from other HCPs: *“[The patient was leaving the ED] to use again and we were completely fine and neutral about that. Something [the patient] may or may not get from health care providers. If [the patient] shares that much [they may be told], ‘But are you sure you’re going to go back [to using drugs] and then tomorrow you might be back here?’ Like there was never even anything close to that when we were having conversations with patients” [Peer 5].*

In equipping patients to lead their own care resourcing, the capacity to effectively translate the variety of supports available was critical to fostering informed consent. Two Peer Workers explicitly noted the efficacy of using accessible language when communicating with patients. Accessible language feels approachable and friendly to the patient, as opposed to medical jargon which can make patients feel alienated: *“Clinicians talk a lot of stuff that people don’t understand. We tend to really focus on being clear and transparent in, ‘Did you understand what I just said? [Can] you tell me what I said to you?’” [Peer 7].*

One peer explained how they extend their translations of medical jargon to translations of community program brochures when nearing discharge to help patients choose next steps and set their expectations: *“If [the patient] needs any additional support whether it be around clinic or any detox beds or anything like that… I explain it first before allowing them to read so they just… know what they are walking into or how to prepare themselves for what’s coming” [Peer 4].* Lastly, Peer Workers explicitly centered the patients in their care resourcing by asking for direct permission to share information or proceed with a referral, noting that: *“…if we feel it’s necessary or we judge for some reason it is appropriate, and the client consents us to share with the team, then we would come back to their nurse and share” [Peer 5].*

#### Theme 3: support for patient preferences on harm reduction

Many Peer Workers viewed their care role as holistic and compassionate, effectively bridging a gap in the biomedical care model. This namely pertained to acknowledging and reflecting the patient’s humanity in their circle of ED care. One Peer Worker described the sterile nature of clinical care as lacking: *“…human contact, right out of that emotional, spiritual, humanity… Our systems are very, very, very, kind of diagnostic and clinical. They’re not really very well rounded. It’s a very linear system. It’s why peer support gives us multi-dimensional levels of presenting ourselves; right, hence we get their attention” [Peer 7].* This Peer Worker pointed to the patients’ overwhelmingly positive reception to their role as proof of there being a historically unmet need that is now being met*: “Once we displayed our own vulnerability, our own humanity, everything starts to just flow. They’ve got to find the human contact” [Peer 7].*

The theme of non-judgment was also woven throughout descriptions of listening to and advocating for the patient’s care preferences. One peer recounted practicing non-judgment by not assuming patients were seeking abstinence or any particular type of support: *“I’m like, ‘Whatever journey you want to take, I’ll support you through and I’ll get you the resources that you need whether it’s harm reduction, if it’s abstinence” … I don’t push because some of them don’t even want to do either one of them… I’m all for whatever that patient wants… I know that getting clean and sober is not for everybody and it’s a journey” [Peer 3].*

Peer Workers recapitulated scenarios in which harm reduction perspectives were key to reflecting the patient’s humanity and advocating for their needs. Harm reduction practices were seen as sometimes lacking in the hospital setting, particularly with respect to addressing the patients’ withdrawal needs. Referencing the all-consuming and difficult experience of withdrawal, one Peer Worker explained how alleviating withdrawal symptoms quickly and easily was a major priority for many patients, yet this prioritization was rarely reflected in the care plans being developed by the clinical team. A Peer Worker reflected that when this incongruence persisted and the patient’s priority need remained unmet, patients often left the ED on their own decision, disintegrating all care plans. One peer described how this attention to harm reduction bridged the gap between the HCPs and the patients: *“If we don’t take steps to address the withdrawal first the patient is not going to be here for you to have your plan going through” [Peer 5].* They noted how personal biases factor into the perceptions of certain patients to dictate the standards of care received, observing a frequent and sometimes seemingly automatic denial of medication to help with pain or withdrawal.

#### Theme 4: peer worker role in facilitating self-acceptance and self-defined recovery

Many Peer Workers described the role as contributing to self-acceptance by expelling any shame they had about their lived experience: *“There was a time when I did not have a sense of value to my own lived experience. I didn’t feel like it really mattered to anybody or anything until I became a peer and now that lived experience is a gold mine to help other people… It’s like you’ve been through all of this and it’s for something and you’re able to give back now” [Peer 7].*

Similarly, two Peer Workers described the role as having had a positive effect on their own healing and recovery journey. The Peer Workers alluded to a strengthened sense of power over their own life and a responsibility to provide the best care possible to patients: *“I even suggest coping mechanisms; some meditation, some yoga, maybe just a long walk to clear your head. It’s helped me too because before my mental state was not all together and me coming in and seeing some of the mental patients and the conditions they’re in, I say to myself, ‘I really need to improve on me so I can better assist them.’ …I realized that some of the stuff I’m suggesting to the patients I should try it too… so I’ve started trying it and it’s helped me” [Peer 4].*

Notably, given the relevance of the Peer Workers’ lived experience, the stories that patients share can certainly be emotionally draining and even triggering. As such, one Peer Worker noted the importance of being in a healthy stage of their recovery to be able to perform the Peer Worker role: “*If you’re not recovered… and you’re not at amends with your own lived life experience, you’re not going to be able to help the patients at the hospital. There’s no way because the stories will be triggering. The stories will bring back a lot of memories, but if you’re okay with yourself and you know you’re okay with your progress and where you are, then you won’t have an issue, but I don’t think anybody who’s not really there would be able to do that. Especially on a daily basis and be able to actually help as much as they would want to” [Peer 2].*

#### Theme 5: importance of supports and resources to help Peer Workers navigate the emotional intensity of the ED setting

Peer Workers referred to the ED as a space where patients are most vulnerable in their journey and often at their lowest points. This presented challenges unique to the ED setting, namely with respect to the intensity of the situations encountered. The weight of facing these situations was compounded by the brevity of the interaction, given that Peer Workers are unable to follow-up with patients long-term. Peer Workers also noted several challenges with broader system-level issues that impeded their ability to provide the best possible care to patients. For example, Peer Workers recounted issues in finding a shelter bed in a timely manner: “*…when you call [the housing phone line] it’s at least 40 min before you get through to somebody that’s going to work with you to try and get that patient a shelter bed…And then when you finally get through and they’re looking on their end and you have to call back in an hour cause there’s nothing available***”***[Peer 1].*

Several Peer Workers interviewed were previously employed at COVID Recovery and Isolation sites for individuals who were infected with COVID and were unhoused. Peer Workers compared the intensity of the ED setting during the interviews, noting the contrast in the environment and situations encountered. The first peer reflected on their naiveté for thinking that the ED role would be the same as their first assignment: *“…you think you already know ‘cause I worked at [the COVID Recovery and Isolation site] before… You think you see it all and then you go to the hospital and you’re like, ‘Oh, this is… new’” [Peer 2].* This peer described their first week in the ED as a fast and intense exposure to a myriad of situations followed by a quick desensitization: “*Within the week you hear every situation possible. Within the first month you’re just like, ‘Yep, I’ve heard this 20 times’… After that it’s nothing” [Peer 2].*

Similarly, drawing from their experience as a Peer Worker at the COVID Recovery and Isolation site, another Peer Worker described the ED setting as: “*Much more intense, and raw and just difficult, harder to process. At the end of the day, I remember I would have to go home sometimes and cry just to release because it was difficult to hold the hand of someone while they’re desperately looking for somewhere else out of their addiction. Their body was telling them to leave right away but they wanted to be there and get treated, but they just cry like a baby, [are] desperate, and in pain… [Patients] remain with me in a way that’s really hard to put in words… A pain for life that I will take, I think—all those interactions I got in the ED” [Peer 5].*

The situations that Peer Workers uncounted elicit a deep sense of care and compassion. Some Peer Workers expressed frustration with the inability to follow-up with patients once they are discharged from the ED, specifically when patients were experiencing a mental health crisis. One Peer Worker described this experience as: “*…frustrating how we don’t get to follow up later to see or to try to connect them and make sure this person gets further support after discharge from the ED” [Peer 5].*

## Discussion

This concurrent mixed-methods evaluation found that Peer Workers promoted patient trust amongst the broader health care team and advocated for shared decision making and patient preferences. Quantitative and qualitative data were combined to comprehensively characterize the role that Peer Workers play, specifically in the supports provided to patients, conflict de-escalation and in encouraging the patient to share information with the health care team. Qualitative data emphasized how Peer Workers contribute to improvements in care transitions, particularly during the discharge period, and how they created space for a trauma-informed approach to care delivery.

Peer Workers shared that their role was instrumental in their path to recovery and self-acceptance but was not without its challenges. Peer Workers described the ED environment as “intense”, “raw”, and “difficult” when comparing the setting to previous environments that they have been employed in as a Peer Worker. Despite this, Peer Workers felt supported by their organization, through the provision of both formal and informal resources and supports.

Our work is largely consistent with the small body of literature available on Peer Workers in hospital-based settings [33–35]. Notably, our findings drew many parallels with that of Collins and colleagues [33], who investigated essential qualities and functions of Peer Workers for people with substance use in the United States. Echoing our findings, the authors found that Peer Workers fostered meaningful connections with patients, built on trust and shared power, translated complex information from the care team in a way that resonated with patients, and helped to bridge established trust with other HCPs [33]. Our findings also re-enforced that Peer Workers play an important role encouraging patients to follow through with treatment plans and in conflict de-escalation [33]. To further investigate these benefits, future research may seek to quantify these impacts on health care use and patient experiences.

Unhoused patients frequently experience poor discharge planning, with discharge to the streets being a common occurrence [44–46]. We found that Peer Workers frequently arranged housing and transportation for patients to support their transition back into the community. This represents a relatively unique feature of this program in that the supports offered acknowledge the structural housing vulnerabilities faced by many patients. Despite the importance of this support, many Peer Workers stressed the difficulty faced when trying to connect patients with emergency housing, citing long wait times and capacity issues. Thus, while patients stand to benefit from receiving support with discharge planning, there ultimately remains a systemic housing issue much larger than any peer role or single hospital can remedy. Even when unhoused patients are successfully discharged to shelters, the provision of follow-up care and an environment that is supportive of a harm reduction approach is lacking [47]. Long-term solutions to this issue necessitate a whole-of-government approach that addresses the root causes of homelessness through the provision of adequate subsidized and supportive housing [48].

Recent literature on the impact of COVID-19 on people with substance use have called for greater supports that address the intersectional effects of being unsheltered and substance use [15], including increased access to social supports and harm reduction services [11]. As part of this program evaluation, we observed that Peer Workers played a vital role in connecting patients with housing and harm reduction supports, which should be considered as part of a larger set of public health and policy strategies for overdose prevention.

Strengths of this study lay in the methodological rigour of the mixed-methods approach. Specifically, we applied principles of credibility, transferability, dependability, confirmability, and reflectivity throughout data collection and analysis [49, 50]. First, we conducted the thematic analysis with two coders to help minimize bias. We conducted the first three interviews with a second interviewer present, which were then followed by peer debriefing and reflexive note-taking throughout data collection, analysis, and interpretation [49]. To enhance the credibility of our findings, we conducted member checking [50]. The qualitative interviews yielded rich descriptions that coloured the experiences of Peer Workers and reinforced quantitative data. Our purposive sampling approach ensured that we interviewed Peer Workers who were employed at both program sites at the hospital. While our sample was modest, the program only employs a small number of Peer Workers at any given time, with our sampling representing over 75% of all Peer Workers employed at the two program sites.

The evaluation findings should be interpreted in consideration of certain limitations. First, this program operates in the context of philanthropic funding within Canada’s publicly funded universal healthcare system, and therefore some of the insights and learnings may not translate directly to Peer Worker programs in other health systems. Comparability of our findings should be considered in light of the similarities/differences in program design and populations served. Although the purpose of this work was to describe how Peer Workers perceive their impact on the patient’s care experience, it is possible that patients and HCPs may have differing views, and therefore speaking with patients and HCPs is an important next step to understand the full benefits of the program. Future work may also seek to understand the long-term impacts of Peer Support on future health care utilization. Additionally, the data collected through the Patient Interaction Survey is reported by a proxy (i.e., the Peer Worker) and is not self-reported by the patient, therefore we were unable to directly ascertain specific patient circumstances (e.g., housing status, gender). Due to a relatively small sample size and the primary aim of our research, we were unable to conduct a comprehensive sex and gender analysis or draw specific inferences based on these factors [51]. Information recorded in the Patient Interaction Survey is limited to that which is known to the Peer Worker (i.e., if the patient disclosed that information), therefore due to the stigma associated with drug use, we anticipate this proportion to be under-reported.

## Conclusions

Our concurrent mixed-methods evaluation of a Peer Worker program in the ED adds to the sparce literature on the impact of Peer Workers on the care of marginalized populations in hospital-based settings. Our findings complement evidence from North America which suggest Peer Workers are a major asset to health care teams. Through disclosing their lived experience, established neutrality from HCPs, and the provision of empathic, humanizing care, Peer Workers can help counteract many of the social and structural barriers that marginalized populations face during interactions with the health care system. In addition to the insights and learnings from this evaluation, our work highlights key areas for future research that may help further refine how Peer Worker interventions are designed to better support marginalized populations.

### Supplementary Information

**Additional file 1. **Patient Interaction Survey.

**Additional file 2. **Peer Worker Qualitative Interview Guide.

## Data Availability

The data generated and analysed during the current study are available from the corresponding author on reasonable request and with Research Ethics Boards approval.

## Abbreviations

COVID-19: Coronavirus disease 2019
ED: Emergency department
HCPs: Health care providers

## References

[1] Hwang SW, Ueng JJM, Chiu S, Kiss A, Tolomiczenko G, Cowan L (2010). Universal health insurance and health care access for homeless persons. Am J Public Health.

[2] Argintaru N, Chambers C, Gogosis E, Farrell S, Palepu A, Klodawsky F (2013). A cross-sectional observational study of unmet health needs among homeless and vulnerably housed adults in three Canadian cities. BMC Public Health.

[3] Urbanoski K, Inglis D, Veldhuizen S (2017). Service Use and Unmet needs for Substance Use and Mental disorders in Canada. Can J Psychiatry.

[4] Kurdyak P, Gandhi S, Holder L, Rashid M, Saunders N, Chiu M (2021). Incidence of Access to Ambulatory Mental Health Care Prior to a Psychiatric Emergency Department visit among adults in Ontario, 2010–2018. JAMA Netw Open.

[5] Kerr T, Wood E, Grafstein E, Ishida T, Shannon K, Lai C (2004). High Rates of Primary Care and Emergency Department Use among Injection Drug users in Vancouver. J Public Health.

[6] Thakarar K, Morgan JR, Gaeta JM, Hohl C, Drainoni M-L (2015). Predictors of frequent emergency room visits among a Homeless Population. PLoS One.

[7] Hwang SW, Chambers C, Chiu S, Katic M, Kiss A, Redelmeier DA (2013). A comprehensive assessment of health care utilization among homeless adults under a system of universal health insurance. Am J Public Health.

[8] Holland KM, Jones C, Vivolo-Kantor AM, Idaikkadar N, Zwald M, Hoots B (2021). Trends in US Emergency Department Visits for Mental Health, Overdose, and Violence outcomes before and during the COVID-19 pandemic. JAMA Psychiatry.

[9] Myran DT, Hsu AT, Smith G, Tanuseputro P (2019). Rates of emergency department visits attributable to alcohol use in Ontario from 2003 to 2016: a retrospective population-level study. CMAJ.

[10] Government of Canada. Opioid and stimulant-related harms in Canada 2021. Available from: https://health-infobase.canada.ca/substance-related-harms/opioids-stimulants/graphs?index=477.

[11] Ontario Drug Policy Research Network, Office of the Chief Coroner for Ontario/Ontario Forensic Pathology Service, Public Health Ontario, Centre on Drug Policy Evaluation. Preliminary patterns in circumstances surrounding opioid-related deaths in Ontario during the COVID-19 pandemic. 2020. Available from: https://www.drugsandalcohol.ie/33326/1/Opioid-Death-Report_FINAL-2020NOV09.pdf.

[12] Gomes T, Kitchen SA, Murray R (2021). Measuring the Burden of opioid-related mortality in Ontario, Canada, during the COVID-19 pandemic. JAMA Netw Open.

[13] Appa A, Rodda LN, Cawley C, Zevin B, Coffin PO, Gandhi M (2021). Drug Overdose deaths before and after shelter-in-place orders during the COVID-19 pandemic in San Francisco. JAMA Netw Open.

[14] Faust JS, Du C, Mayes KD, Li S-X, Lin Z, Barnett ML (2021). Mortality from drug overdoses, homicides, unintentional injuries, Motor Vehicle Crashes, and Suicides during the pandemic, March-August 2020. JAMA.

[15] Liu M, Richard L, Campitelli MA, Nisenbaum R, Dosani N, Dhalla IA et al. Drug overdoses during the COVID-19 Pandemic among recently homeless individuals. Addiction. 2022. 10.1111/add.15823 PMC9111216 35129239

[16] Myran DT, Cantor N, Pugliese M, Hayes T, Talarico R, Kurdyak P (2021). Sociodemographic changes in emergency department visits due to alcohol during COVID-19. Drug Alcohol Depend.

[17] Englander H, Collins D, Perry SP, Rabinowitz M, Phoutrides E, Nicolaidis C (2018). We’ve learned it’s a medical Illness, not a Moral choice: qualitative study of the effects of a Multicomponent Addiction Intervention on Hospital Providers’ attitudes and experiences. J Hosp Med.

[18] McNeil R, Small W, Wood E, Kerr T (2014). Hospitals as a ‘risk environment’: an ethno-epidemiological study of voluntary and involuntary discharge from hospital against medical advice among people who inject Drugs. Soc Sci Med.

[19] Choi M, Kim H, Qian H, Palepu A (2011). Readmission rates of patients discharged against medical advice: a matched cohort study. PLoS One.

[20] Hwang SW, Weaver J, Aubry T, Hoch JS (2011). Hospital costs and length of stay among homeless patients admitted to medical, surgical, and psychiatric services. Med Care.

[21] Khorasheh  TKG, Kenny K (2022). Experiences of overdose among people who inject drugs in the City of Toronto.

[22] Velez CM, Nicolaidis C, Korthuis PT, Englander H (2017). It’s been an experience, a life learning experience: a qualitative study of hospitalized patients with Substance Use disorders. J Gen Intern Med.

[23] Skosireva A, O’Campo P, Zerger S, Chambers C, Gapka S, Stergiopoulos V (2014). Different faces of discrimination: perceived discrimination among homeless adults with mental Illness in healthcare settings. BMC Health Serv Res.

[24] Ti L, Tzemis D, Buxton JA (2012). Engaging people who use Drugs in policy and program development: a review of the literature. Subst Abuse Treat Prev Policy.

[25] Repper J, Carter T (2011). A review of the literature on peer support in mental health services. J Mental Health.

[26] Sunderland K, Mishkin W. Guidelines for the practice and training of peer support. Mental Health Commission of Canada; 2014.

[27] Irestig R, Burström K, Wessel M, Lynöe N (2010). How are homeless people treated in the healthcare system and other societal institutions? Study of their experiences and trust. Scand J Public Health.

[28] Mills ED, Burton CD, Matheson C (2015). Engaging the citizenship of the homeless—a qualitative study of specialist primary care providers. Fam Pract.

[29] Carey C, Jones R, Yarborough H, Kahler Z, Moschella P, Lommel K (2018). 366 peer-to-peer addiction counseling initiated in the Emergency Department Leads to high initial opioid recovery rates. Ann Emerg Med.

[30] Reif S, Braude L, Lyman DR, Dougherty RH, Daniels AS, Ghose SS (2014). Peer recovery support for individuals with substance use disorders: assessing the evidence. Psychiatr Serv.

[31] Waye KM, Goyer J, Dettor D, Mahoney L, Samuels EA, Yedinak JL (2019). Implementing peer recovery services for Overdose prevention in Rhode Island: an examination of two outreach-based approaches. Addict Behav.

[32] Bassuk EL, Hanson J, Greene RN, Richard M, Laudet A (2016). Peer-Delivered Recovery Support Services for Addictions in the United States: a systematic review. J Subst Abuse Treat.

[33] Collins D, Alla J, Nicolaidis C, Gregg J, Gullickson DJ, Patten A, et al. If it wasn’t for him, I wouldn’t have talked to them: qualitative study of addiction peer mentorship in the hospital. J Gen Intern Med. 2019;34:1–8. 10.1007/s11606-019-05311-0 31512181

[34] Englander H, Gregg J, Gullickson J, Cochran-Dumas O, Colasurdo C, Alla J (2020). Recommendations for integrating peer mentors in hospital-based addiction care. Subst Abus.

[35] Lennox R, Lamarche L, O’Shea T (2021). Peer support workers as a bridge: a qualitative study exploring the role of peer support workers in the care of people who use Drugs during and after hospitalization. Harm Reduct J.

[36] University Health Network. Report to our community. 2019. Available from: https://www.uhn.ca/corporate/AboutUHN/Documents/report-to-our-community-2018-19.pdf.

[37] Earheart J, Crisanti A (2019). Peer support workers in the ED: a Report. Division of Community and Behavioural Health. Department of Psychiatry and Behavioural Sciences.

[38] Salzer MS, Shear SL (2002). Identifying consumer-provider benefits in evaluations of consumer-delivered services. Psychiatr Rehabil J.

[39] Jull J, Giles A, Graham ID (2017). Community-based participatory research and integrated knowledge translation: advancing the co-creation of knowledge. Implement Sci.

[40] Creswell JW, Clark VLP. Designing and conducting mixed methods research. Thousand Oaks: Sage Publications; 2017.

[41] Patton M (2002). Qualitative Research & Evaluation Methods.

[42] Braun V, Clarke V (2006). Using thematic analysis in psychology. Qual Res Psychol.

[43] Tong A, Sainsbury P, Craig J (2007). Consolidated criteria for reporting qualitative research (COREQ): a 32-item checklist for interviews and focus groups. Int J Qual Health Care.

[44] Gaetz S (2017). A new direction: a framework for homelessness prevention.

[45] Backer TE, Howard EA, Moran GE (2007). The role of effective discharge planning in preventing homelessness. J Prim Prev.

[46] Jenkinson JIR, Strike C, Hwang SW, Di Ruggiero E (2021). Nowhere to go: exploring the social and economic influences on discharging people experiencing homelessness to appropriate destinations in Toronto, Canada. Can J Public Health.

[47] Canham SL, Custodio K, Mauboules C, Good C, Bosma H (2020). Health and Psychosocial needs of older adults who are experiencing Homelessness following Hospital Discharge. Gerontologist.

[48] Baral S, Bond A, Boozary A, Bruketa E, Elmi N, Freiheit D (2021). Seeking shelter: homelessness and COVID-19. FACETS.

[49] Korstjens I, Moser A (2018). Series: Practical guidance to qualitative research. Part 4: trustworthiness and publishing. Eur J Gen Pract.

[50] Lincoln Y, Guba EG (1985). Naturalistic inquiry.

[51] Heidari S, Babor TF, De Castro P, Tort S, Curno M (2016). Sex and gender equity in research: rationale for the SAGER guidelines and recommended use. Res Integr Peer Rev.

