# Supplementary material for: “Whatever journey you want to take, I’ll support you through”: a mixed methods evaluation of a peer worker program in the hospital emergency department
Source: BMC Health Serv Res. 2024 Jan 30;24:147. doi: 10.1186/s12913-023-10532-5 (PMC10826204; doi:10.1186/s12913-023-10532-5)
Supplement: Supplementary file 1 — Additional file 1. Patient Interaction Survey. [file 12913_2023_10532_MOESM1_ESM.docx]

**Additional File 1.** Patient Interaction Survey

1. **Emergency department location:**

- Toronto Western Hospital
- Toronto General Hospital

1. **Gender of patient:**

- Man
- Woman
- Other (please specify)

1. **Age group of patient:**

- Under 18 years
- 18 to 29 years
- 30 to 39 years
- 40 to 49 years
- 50 to 59 years
- 60 to 69 years
- 70 to 79 years
- 80 years or older

1. **What was the main reason for the Emergency Deparment visit?** (select all that apply)

- Mental heath-related issue
- Substance use-realted issue
- Suspected COVID-19 case (e.g., patient recieved a COVID-19 test)
- Injury
- General weakness or pain
- Medicalissue
- Personal issue (e.g., emotional crisis)
- Another reason (please specify)

1. **Please specify the substance(s) the patient stated they have used**

- Alcohol
- Downers (e.g., opioids, sedatives, benzodiazepines ect.)
- Uppers (e.g., methamphetamine, cocaine ect.)

1. **What service referrals did you offer to the patient.?** (Select all that apply)

*Note: a referral can include a formal call to a centre, or informal sharing of information such as providing directions to a centre or providing a brochure.*

- Connected patient with their The Neighbourhood Group case worker
- Rapid Access Addiction Medicine Clinic, detox/rehab centre, or other addiction support group (e.g., Alcoholics anonymous, Narcotics anonymous)
- Mental health treatment centre
- Other treatment centre (please specify)
- COVID-19 isolation site
- Shelter bed
- Long-term housing support (e.g., help with housing application)
- Employment support
- Income aid (e.g., help with Ontario Works application)
- Hot meal/food site
- Call to crisis hotline (e.g., domestic violence)
- Not listed (please specify)
- No referrals were provided

1. **If applicable, did you physically accompany the patient to any of the above referrals?** *(For example, did you walk them to the site?)*

- Yes
- No
- Not applicable

1. **What tangible resources did you offer the patient?** *Select all resources that were offered, regardless of whether the patient accepted or declined.*

- Naloxone kit
- Other harm reduction supplies (e.g., long needles)
- Clothing
- TTC tokens/taxi transport
- Food
- Not listed (please specify)
- No tangible resources were offered

1. **Did the patient accept any of the tangible resources offered?**

- Yes, all of them
- Yes, some of them
- No, none of them

1. **Did you or another staff member at the Emergency Department provide a Naloxone kit?**

- I did
- Another staff member did

1. **What date did you provide the patient with the Naloxone kit?**
2. **Did you provide the client with an explanation of what Naloxone is used for and how to use it?**

- Yes
- No

1. **How else did you support the patient?**

- Had friendly & empathetic conversations
- Shared lived experience
- Helped navigate their emotions/mental wellbeing
- Supported discharge planning
- Advocated to hospital staff for patient care
- Provided information no hospital resources or what will be happening in the Emergency Department
- Brought them out for a cigarette break
- Not listed (please specify)

1. **Did you share new information about the patient with other ED staff/care providers?** *(For example, with a nurse, physician, social worker ect.)*

- Yes
- No

1. **Please provide a short written description of how you supported this individual**
2. **How did the patient leave the Emergency Department?**

- Patient was escorted out by security
- Patient left before they were discharged and they were visibly agitated/distressed
- Patient left before they were discharged
- Patient left at the right time
- Patient left at the right time and expressed thanks
- Patient left at the right time and said they would be back tot his hospital next time they needed care
- Not applicable (e.g., the patient was admitted or you don’t know how/when the patient left the ED)

1. **Did the patient begin to escalate during their stay?**

*Escalation refers to increasing intensity or seriousness of a situation (for example, an upset patient can escalate to yelling or violence)*

*De-escalation refers to decreasing the intensity or seriousness of a situation (for example, calming down a visibly angry or violent patient)*

- Yes
- No
- Don’t know

1. **If you were aware of the escalation while it was happening, were you able to help de-escalate the patient?**

- Yes
- No
- Not applicable- I only found out about the escalation after it already happened

1. **Please describe the tools and/or approaches did you use to help de-escalate the patient?** *(For example, took them out for a cigarette break, had one-on-one discussion ect.)*
2. **Was security also present for the escalation?**

- Yes, security took control of the situation
- Yes, security was present, but the Peer Workers managed the situation
- No, the Peer Workers managed the situation

1. **Did the patient express wanting to leave before being formally discharged?**

- Yes, and I was aware while they were still in the Emergency Department
- Yes, but I was only made aware once they had left
- No

1. **Did you support the patient in continuing their Emergency Department stay?**

- Yes, I supported them and they still left
- Yes, I supported them and they stayed
- No, I didn’t support them
