# Supplementary material for: “Whatever journey you want to take, I’ll support you through”: a mixed methods evaluation of a peer worker program in the hospital emergency department
Source: BMC Health Serv Res. 2024 Jan 30;24:147. doi: 10.1186/s12913-023-10532-5 (PMC10826204; doi:10.1186/s12913-023-10532-5)
Supplement: Supplementary file 2 — Additional file 2. Peer Worker Qualitative Interview Guide. [file 12913_2023_10532_MOESM2_ESM.docx]

**Additional File 2:** Peer Worker Qualitative Interview Guide

1. I would like to begin by asking you some questions about your experiences with providing peer support to patients in the emergency department. To start, can you describe for me what a typical day looks like for you as a peer support worker in the Peer Support in ED Program?
   1. When does your shift typically start? When does it typically end?
   2. How often do you interact with patients on average during their stay/each day?
   3. Did you work in the peer program at one or both hospital sites?
2. Can you tell me about your experiences with listening to patients?
   1. Can you share any examples of how you responded to a patient’s needs?
   2. Can you share any examples of how you provided comfort to a patient?
   3. Were there situations that you found particularly challenging when listening and interacting with patients?
   4. Would the patient share information with you that they wouldn’t share with the medical team?
3. Can you tell me about your experiences with communication about patients’ preferences or needs to health care providers or hospital staff in the ED?
   1. When communicating the patient’s needs, what works well? What doesn’t work?
   2. Do you feel that there are any issues around the communication and information sharing about patients’ preferences or needs with health care providers or hospital staff that need to be addressed?
   3. Have these relationships with health care providers changed over time? If so, how?
4. In your opinion, how have patients benefitted from the Peer Support in ED Program?
   1. How, if at all, has peer support helped patients adhere to the medical plan outlined by the patients care team?
   2. How has peer support in the ED helped to address patient needs related to accessing social supports?
   3. How has peer support in the ED helped to address patient needs related to their mental health or substance use?
   4. How has peer support helped to address patient needs related to social connections?
5. In your opinion, how has working as a peer support worker in the ED impacted your life?
   1. How has the Peer Support in ED Program impacted your mental health or wellbeing, if at all?
   2. How has the Peer Support in ED Program impacted your work skills or work experience for future employment, if at all?
6. Do you have any recommendations for what can be improved in the Peer Support in ED Program?
   1. Based on your experiences, what advice would you give to someone who was interested in working as a peer worker in the ED?
7. Is there anything else that you would like to share with us related to your experiences with the Peer Support in ED Program that we haven’t discussed today?
